# Dynamic AFM detection of the oxidation-induced changes in size, stiffness, and stickiness of low-density lipoprotein

**DOI:** 10.1186/s12951-020-00727-x

**Published:** 2020-11-12

**Authors:** Kun Wang, Yuanfang Li, Chao Luo, Yong Chen

**Affiliations:** 1 Jiangxi Key Laboratory for Microscale interdisciplinary Study, Institute for Advanced Study, Nanchang University, Nanchang, 330031 Jiangxi P. R. China; 2 School of Materials Science and Engineering, Nanchang University, 999 Xuefu Ave., Honggutan District, Nanchang, 330031 Jiangxi P. R. China

**Keywords:** Low-density lipoprotein (LDL), Atherosclerosis, Atomic force microscopy (AFM), LDL oxidation, Young’s modulus, Adhesion force

## Abstract

**Background:**

Low-density lipoprotein (LDL) is an important plasma lipoprotein transporting lipids to peripheral tissues/cells. The oxidation of LDL plays critical roles in atherogenesis and its oxidized form (oxLDL) is an important risk factor of atherosclerosis. The biomechanical properties of LDL/oxLDL are closely correlated with the disease. To date, however, the oxidation-induced changes in size and biomechanical properties (stiffness and stickiness) of LDL particles are less investigated.

**Methods:**

In this study, copper-induced LDL oxidation was confirmed by detecting electrophoretic mobility, malondialdehyde production, and conjugated diene formation. Then, the topographical and biomechanical mappings of LDL particles before/after and during oxidation were performed by using atomic force microscopy (AFM) and the size and biomechanical forces of particles were measured and quantitatively analyzed.

**Results:**

Oxidation induced a significant decrease in size and stiffness (Young’s modulus) but a significant increase in stickiness (adhesion force) of LDL particles. The smaller, softer, and stickier characteristics of oxidized LDL (oxLDL) partially explains its pro-atherosclerotic role.

**Conclusions:**

The data implies that LDL oxidation probably aggravates atherogenesis by changing the size and biomechanical properties of LDL particles. The data may provide important information for a better understanding of LDL/oxLDL and atherosclerosis.
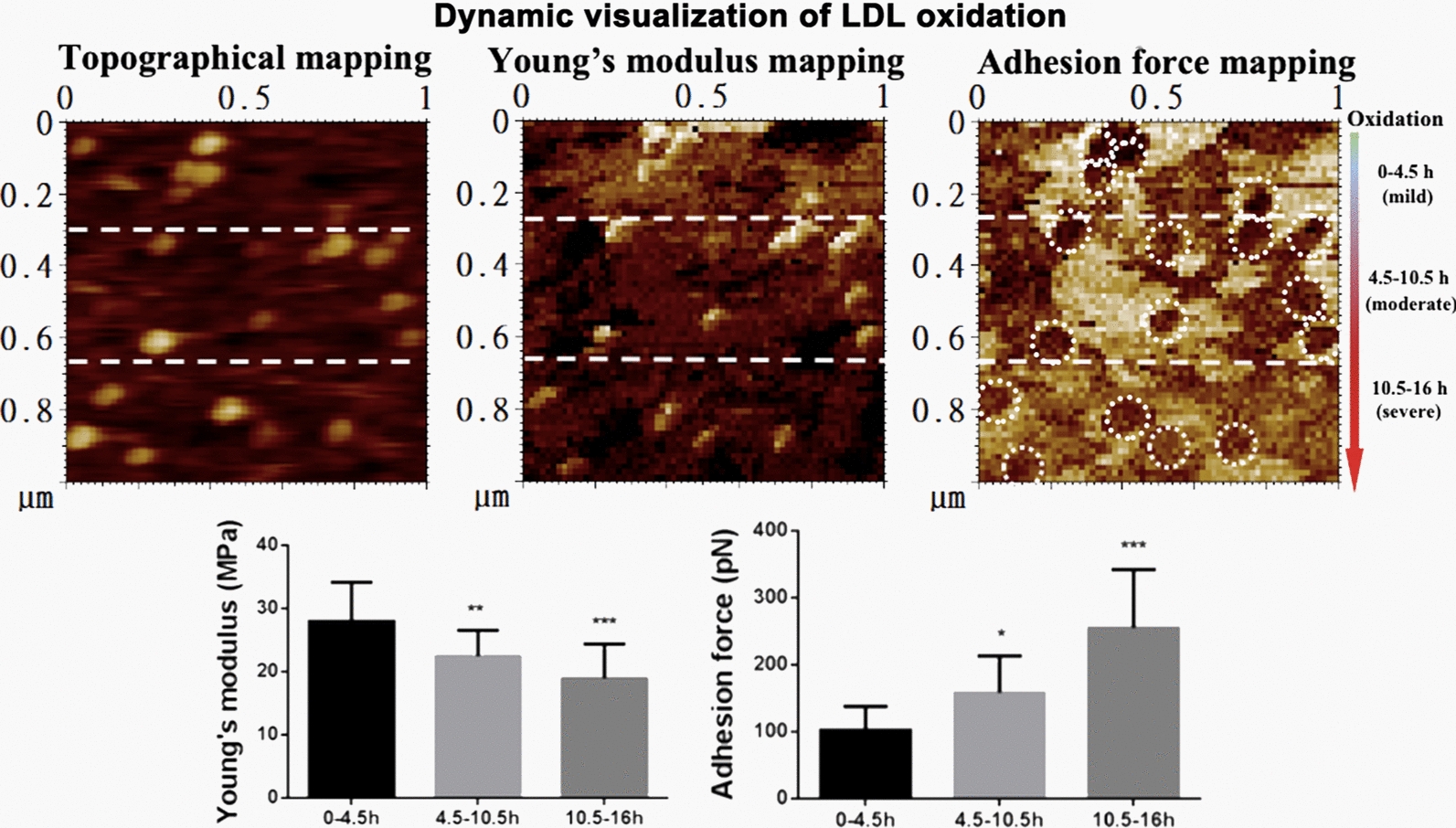

## Background

Low-density lipoprotein (LDL) is one of the five lipid-transporting plasma lipoproteins in human blood circulation. The elevated level of LDL-cholesterol (LDL-C) has been regarded as a major risk factor of atherosclerosis which is a chronic cardiovascular disease characterized by the formation of atherosclerotic plaques and subsequently the narrowing of arterial lumens and is one of the leading causes of death and disability worldwide [1, 2]. The oxidized form of LDL (oxLDL) plays a critical role in the initiation and sustainability of atherogenesis by triggering lipid deposition/accumulation in cells (particularly macrophages) in the subendothelial space (i.e. the intima layer of arterial wall) [3, 4]. We hypothesized that LDL oxidation may also change the physical properties (e.g. size and biomechanics) of LDL/oxLDL particles which will be involved in atherogenesis.

The biochemical/morphological properties and lipid/protein components of native LDL have been well studied. The sizes of LDL particles have also been measured by many techniques including gradient gel electrophoresis (GGE), dynamic light scattering (DLS), high-performance gel-filtration chromatography, transmission electron microscopy (TEM), cryo-electron microscopy, and nuclear magnetic resonance (NMR) as well as atomic force microscopy (AFM) [5]. Until now, however, the biomechanical properties of native LDL are less investigated, and therefore the effects of oxidation on biomechanical properties of LDL are poorly understood.

Besides the imaging (size measurement) function on the nanoscale, force measurement is another major powerful function of AFM which allows the detection of biomechanical properties of nanometer-sized LDL particles. AFM has been widely applied to detect the biomechanical properties or changes of biological samples, such as cells and biomolecules [6, 7]. Unfortunately, however, AFM detection of the biomechanical properties of plasma lipoproteins is poorly applied until now. In this study, we sought to investigate the effects of oxidation on the biomechanical properties (e.g. Young’s modulus and adhesion force) of LDL/oxLDL particles via dynamic AFM detection.

## Methods

### Reagents

Human plasma LDL was purchased from Yiyuan Biotechnologies (Guangzhou, China). *N*,*N*-diisopropylethylamine (DIPEA), aminopropyltriethoxysilane (APTES), and glutaraldehyde for mica functionalization were from TCI (Shanghai, China; or Tokyo, Japan for APTES).

### Copper-induced oxidation of LDL

Prior to LDL oxidation, ethylene diamine tetraacetic acid (EDTA) was removed from LDL solutions via dialysis at 4 °C for 36 h. For oxidation, native LDL solutions were mixed with cupric sulphate (99.0% purity; Jinshanting New Chemical Reagent Factory, Shanghai, China) at a final copper concentration of 5 µM in phosphate-buffered saline (PBS) at 37 °C for indicated periods of time. The oxidation was terminated by 5 µM EDTA. After removing EDTA and cupric sulphate via centrifugation at 10,000×*g* at 4 °C for ~ 15 min by using the Amicon Ultra-0.5 ml, 100 kDa Centrifugal Filter Unit (Merck Millipore), the oxLDL samples were subjected to the following experiments. The concentration of LDL/oxLDL was determined by bicinchoninic acid (BCA) assay and adjusted with sterile PBS.

For the dynamic AFM detection of LDL oxidation, the functionalized mica sheet pre-immobilized with native LDL particles was put on the sample stage of atomic force microscope; after adding the cupric sulphate solution at a final copper concentration of 5 µM in PBS, the LDL/oxLDL particles were imaged by AFM immediately and the imaging lasted for around 16 h.

### Confirmation of LDL oxidation

LDL oxidation was confirmed by detecting electrophoretic mobility via agarose gel electrophoresis, malondialdehyde (MDA) production via TBARS assay kit, and dynamic conjugated diene (CD) formation, respectively as reported previously [8, 9]. The LDL samples were oxidized for the indicated periods of time. The absorbance of each sample at 532 nm and 234 nm for MDA production and CD formation, respectively was detected by an UV-5100 spectrophotometer. The time-vs-response fitting curves were obtained by fitting calculated averages to a four-parameter function using GraphPad Prism 6.0 software.

### Mica functionalization and sample preparation

The mica functionalization and sample preparation were performed via the APTES-glutaraldehyde method as reported in our previous studies [10, 11]. Briefly, freshly cleaved mica sheets in petri dishes were exposed to APTES vapor by keeping the sheets for 2 h with DIPEA and APTES solutions and then for 2 days with DIPEA solution in a sealed glass dessicator (ultrapure argon was used to remove the air and moisture in the dessicator). Subsequently, the APTES-modified mica sheets were treated with 0.2% glutaraldehyde for 10 min, rinsed with double distilled water, incubated with native LDL or oxLDL for 2 h, washed with PBS, incubated with l-glycine for 15 min, rinsed again, and subjected to AFM experiments.

### Atomic force microscopy (AFM)

An Agilent series 5500 AFM (Agilent Technologies, CA) equipped with a scanner of 90 µm × 90 µm × 10 µm was recruited to detect the lipoprotein samples immobilized on the functionalized mica sheets. Silicon nitride (Si_3_N_4_) tips (qp-BioAC; NanoSensors, USA) with an end radius of 10 nm and a spring constant of ~ 0.06 N/m were used. Tapping mode and contact mode were utilized for imaging and force measurement in liquid, respectively. The AFM probe was scanned across the mica surface at 0.5–1 Hz with a tracking force of 300–500 pN. The instrument-equipped software (PicoView 1.14) was utilized to analyze AFM data. All topographical images were flattened by one level. Deconvolution was performed via PicoImage 6.2 (an instrument-equipped software). The height (*h*) and full width at half maximum (FWHM; radius *r* is half of FWHM) were extracted from AFM topographical images based on which the volume (*V*) of a single particle was calculated via the equation *V* = (*π* ⋅ *h*/6) ⋅ (3*r*^2^ + *h*^2^) and the equivalent diameter of a sphere was obtained [11]. After obtaining force-vs-distance curves (force measurement), Young’s modulus mapping and adhesive force mapping were constructed via PicoView 1.14, and the Young’s modulus and adhesive force data of individual particles were extracted from the positions on the biomechanical images (Young’s modulus and adhesive force) corresponding to the positions of the particles on the topographical images as described below in detail. The topographical imaging for size measurement and the topographical mapping obtained together with the biomechanical mappings were performed separately. Prior to AFM imaging of LDL/oxLDL particles, the bare and functionalized mica sheets have been detected in liquid and displayed no particles (data not shown).

For force curve mapping, the AFM tip approached the surface at 200 nm/s, the deflection value of the cantilever increased 0.1–0.3 mV from the deflection before contact, after contacting the sample surface, a loading force of 2 nN was applied, and then the tip retracted from the surface at 200 nm/s. The cantilever spring constant was measured using a thermal K method program which is equipped with the instrument, and the deflection sensitivity of cantilever was calibrated on a mica in PBS buffer before force curve mapping. The Young’s modulus was extracted from the force curves of the approach process via the Hertz model [12] using the instrument-equipped PicoView 1.14 software whereas the adhesion force was extracted from the force curves of the retract process. Individual Young’s modulus and adhesion force maps were composed of 4096 force curves (scan size: 1 µm × 1 µm; resolution: 64 pixel × 64 pixel). For quantification, the Young’s modulus (or adhesion force) of a single LDL/oxLDL particle was calculated as the average value of four pixels in the center of each particle-like patch on the Young’s modulus or adhesion force maps, and the average Young’s modulus (or average adhesion force) of individual LDL/oxLDL particles was calculated from 100 particles.

### Statistical analysis

All values from at least three independent experiments are expressed as mean ± SD in the text and graphs. Statistical analysis was performed using Student *t* test to determine the signification (A value of P < 0.05 was considered statistically significant).

## Results

### Confirmation of LDL oxidation

**Fig. 1:**
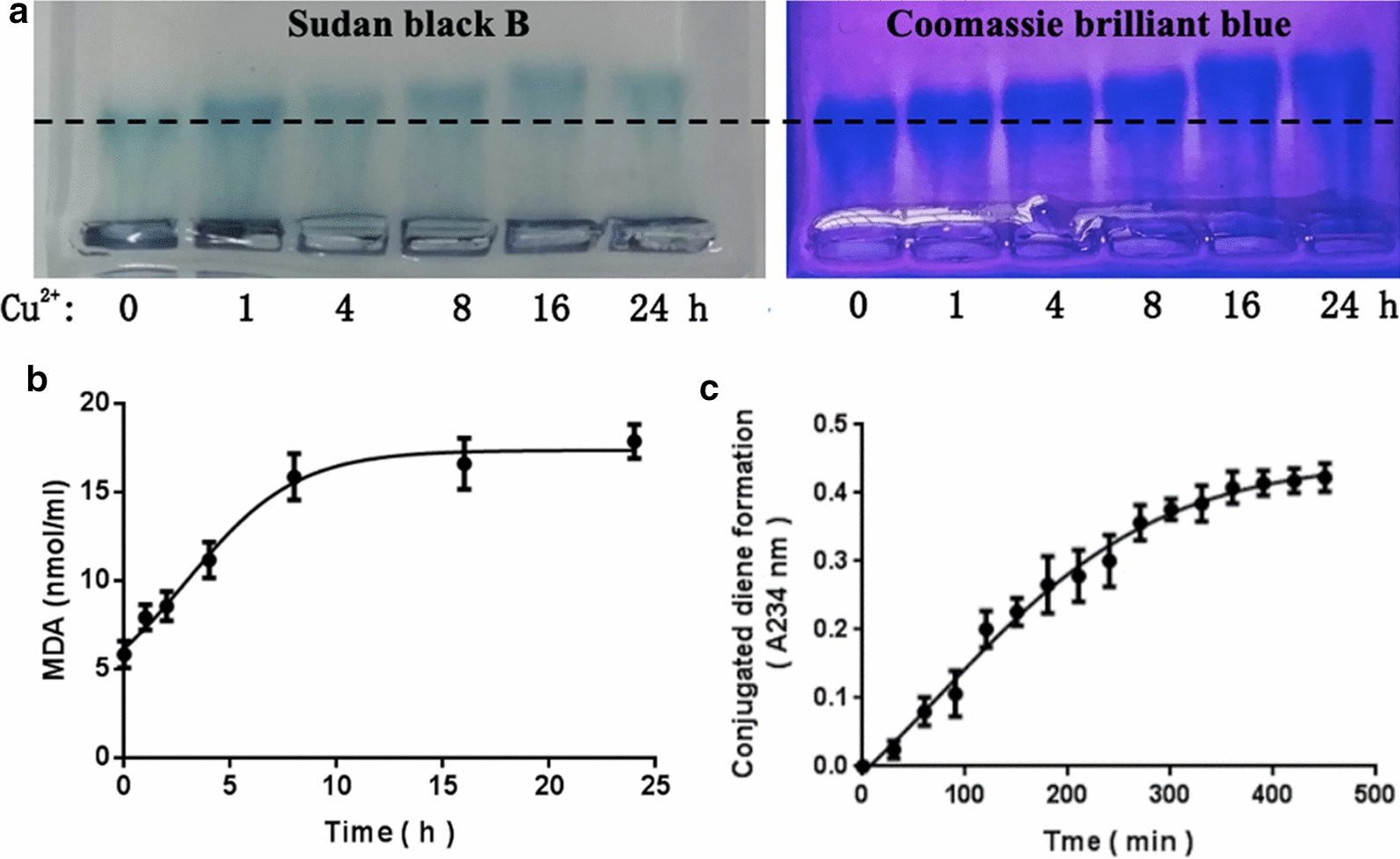
Confirmation of LDL oxidation. **a** Agarose gel electrophoresis. Left panel: the gel stained with Sudan black B for the lipid component of LDL; right panel: the gel loading the same samples but stained with Coomassie brilliant blue for the protein component of LDL. Lanes from left to right: LDL treated with Cu^2+^ (cupric sulphate) at a final copper concentration of 5 µM in PBS for the indicated periods of time. **b** Malondialdehyde (MDA) production of LDL after copper-induced oxidation for the indicated periods of time (h). **c** Conjugated diene formation of LDL after copper-induced oxidation for the indicated periods of time (min). The fitting curves in **b** and **c** were obtained by fitting calculated averages to a four-parameter function

Copper-induced oxidation is the most efficient and most widely used method for in vitro LDL oxidation, and cupric sulphate is an inorganic small molecule which can be easily removed from the LDL/oxLDL solution after oxidation and will not interfere with AFM imaging of LDL/oxLDL particles. Therefore, the copper-induced oxidation was recruited in this study. More negative charges, malondialdehyde (MDA) production, and conjugated diene formation are the major characteristics of LDL oxidation which can be determined by agarose gel electrophoresis, TBARS assay, and spectrophotometer, respectively. Figure 1a shows the electrophoretic migration of native LDL and the LDL treated with cupric sulphate at a final copper concentration of 5 µM for the indicated periods of time on agarose gels. Longer time of copper treatment caused faster migration of LDL, implying severer oxidation of LDL. Figure 1b, c show that longer time of copper treatment caused higher concentration of MDA in 24 h and conjugated dienes within 450 min. All these data imply the success of LDL oxidation.

### Imaging and size measurement of LDL particles before and after oxidation

**Fig. 2:**
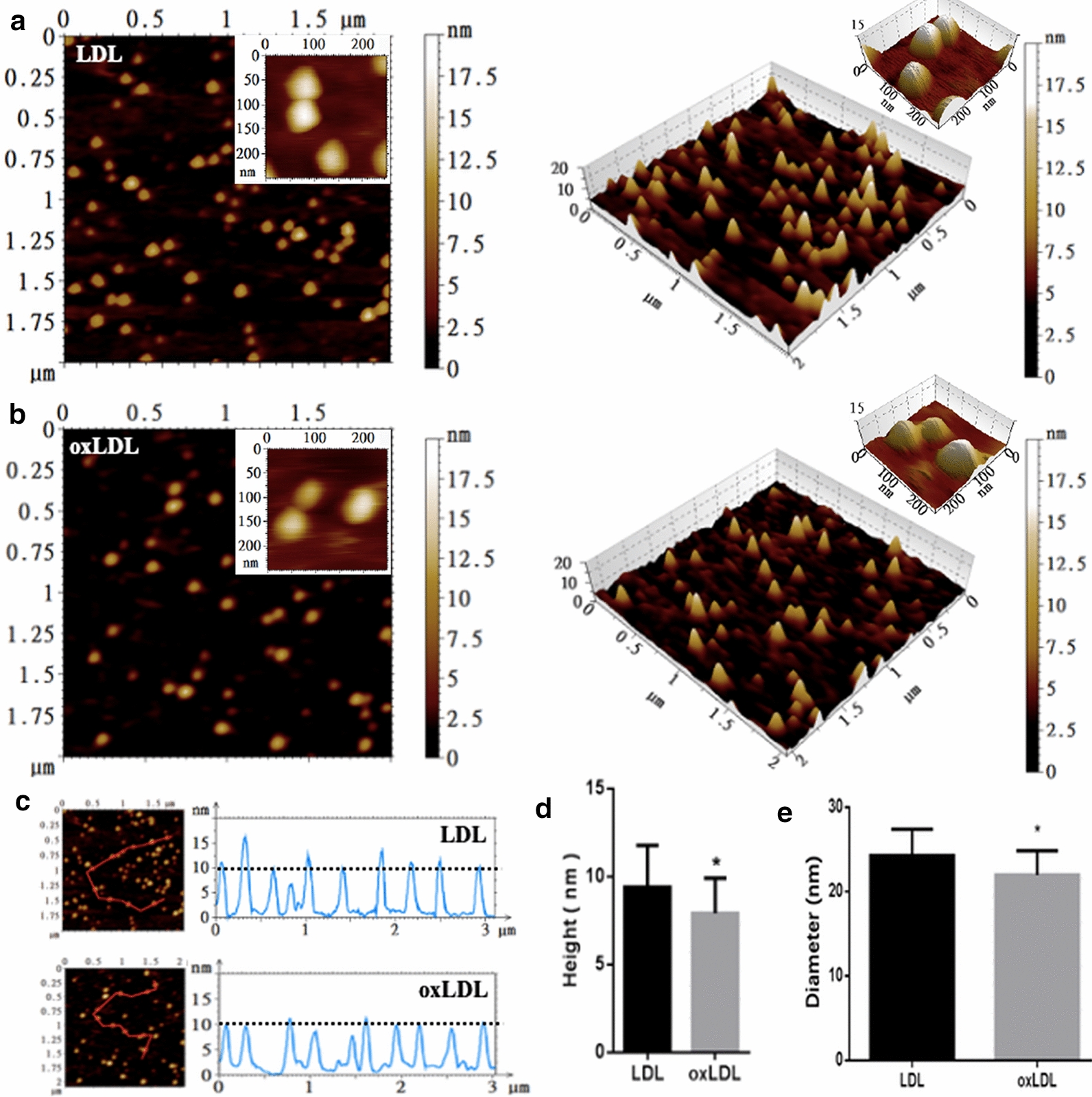
AFM imaging and size measurement of LDL and oxLDL particles in PBS buffer (pH7.4). **a** Representative two dimentional (2-D; left) and 3-D (right) topographical images of LDL particles in a field of 2 µm × 2 µm. **b** Representative 2-D (left) and 3-D (right) topographical images of oxLDL particles in a field of 2 µm × 2 µm. Insets in **a**, **b** representative 2-D (left) and 3-D (right) topographical images of several LDL/oxLDL particles in a field of 250 nm × 250 nm. **c** Height profiles of representative LDL and oxLDL particles. Left panels: the same images as **a** and **b** with a red line across some particles; right panels: the height profiles of the particles. **d** Average height of individual LDL/oxLDL particles (n = 200). **e** Average diameter (i.e. equivalent diameter of a sphere) of individual LDL/oxLDL particles (n = 200). OxLDL particles were the LDL particles oxidized by 5 µM copper at 37 °C for 16 h

Native LDL particles and severely oxidized LDL particles (the LDL oxidized by 5 µM copper at 37 °C for 16 h) were immobilized on the functionalized mica sheets and imaged under the physiological condition (PBS buffer at pH 7.4) by AFM. Figure 2a shows the 2-dimentional (2-D) and 3-D topographical images of native LDL particles and Fig. 2b shows the 2-D and 3-D topographical images of oxLDL particles. Figure 2c shows height profiles of the cross sections across individual LDL and oxLDL particles indicated by the red lines in the topographical images (left panels). It seems that no differences in size between LDL and oxLDL particles can be distinguished by the naked eye only based on the topographical images (Fig. 2a, b). However, the height profiles tell us that LDL particles probably are taller than oxLDL particles (Fig. 2c) which is confirmed by the quantification of average height (9.4 $\pm$ 2.4 nm for LDL vs. 7.9 $\pm$ 1.8 nm for oxLDL; Fig. 2d). Further analysis (Fig. 2e) shows that the average size/diameter of LDL particles (24.1 $\pm$ 2.8 nm) is slightly but significantly larger than that of oxLDL particles (21.9 $\pm$ 2.7 nm), implying that oxidation induced the decrease in size of LDL particles.

### Biomechanical mapping and quantification of LDL particles before and after oxidation

**Fig. 3:**
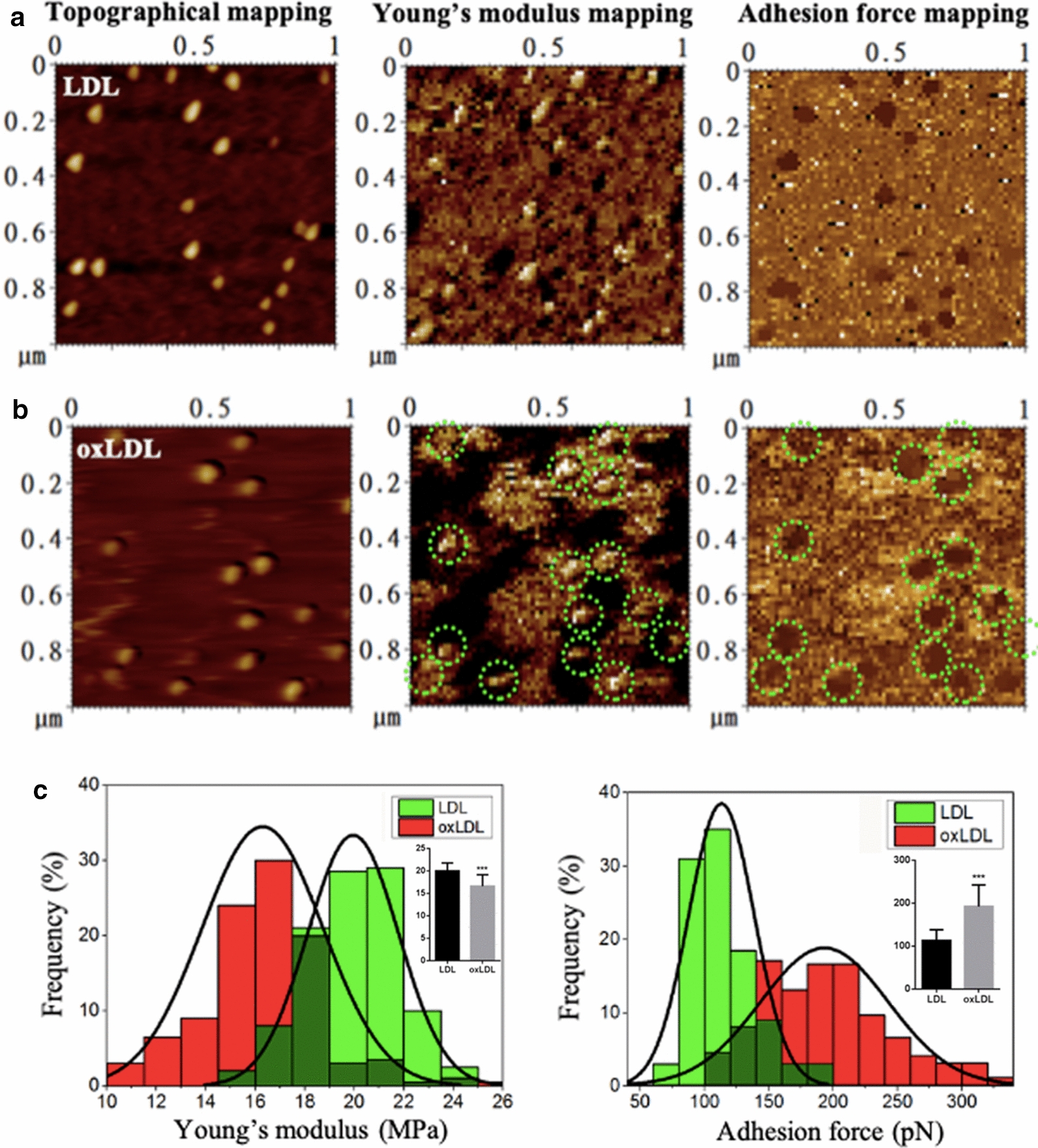
Effects of oxidation on the biomechanical properties of LDL particles. **a** Native LDL particles in a field of 1 µm × 1 µm. **b** Oxidized LDL particles (oxLDL particles; the LDL particles were oxidized by 5 µM copper for 16 h in solution prior to the immobilization on functionalized mica for AFM detection) in a field of 1 µm × 1 µm. **a**, **b** Left panel: topographical mapping; middle panel: Young’s modulus mapping; right panel: adhesion force mapping. Green dotted circles indicate individual particle-like patches corresponding to the oxLDL particles at the same locations in the topographical image. **c** Distribution and quantitative analyses (insets) of Young’s modulus (left panel) and adhesion force (right panel) of individual LDL/oxLDL particles (n = 100; the dark green represents the overlapping part)

The topographical and biomechanical properties of samples can be detected together by AFM. The topographical mapping/imaging, Young’s modulus mapping, and adhesion force mapping were performed in the same field of interest. A little shift of the field might happen between a topographical imaging/mapping and its biomechanical mapping because topographical imaging and force mapping conducted subsequently but not simultaneously. Figure 3a shows the topographical, Young’s modulus, and adhesive force maps of native LDL particles and Fig. 3b shows the mappings of the severely oxidized LDL particles (the LDL oxidized by 5 µM copper at 37 °C for 16 h). Corresponding to the position of each particle on the topographical mapping, there was a particle-like patch obviously different from the surroundings/background on the Young’s modulus or adhesion force mapping. Therefore, the Young’s modulus or adhesion force data of each LDL/oxLDL particle was extracted from the particle-like patch on the Young’s modulus or adhesion force mapping. The quantitative analyses (Fig. 3c) show that the average Young’s modulus of oxLDL particles (16.64 ± 2.56 MPa) was significantly lower than that of native LDL particles (19.98 ± 1.81 MPa) whereas the average adhesion force of oxLDL particles (192.3 ± 50.2 pN) was significantly stronger than that of native LDL particles (113.1 ± 24.9 pN). The data implies that oxidation made LDL particles softer and stickier.

### Dynamic detection of the oxidation-induced changes in biomechanical properties of LDL particles during oxidation

**Fig. 4:**
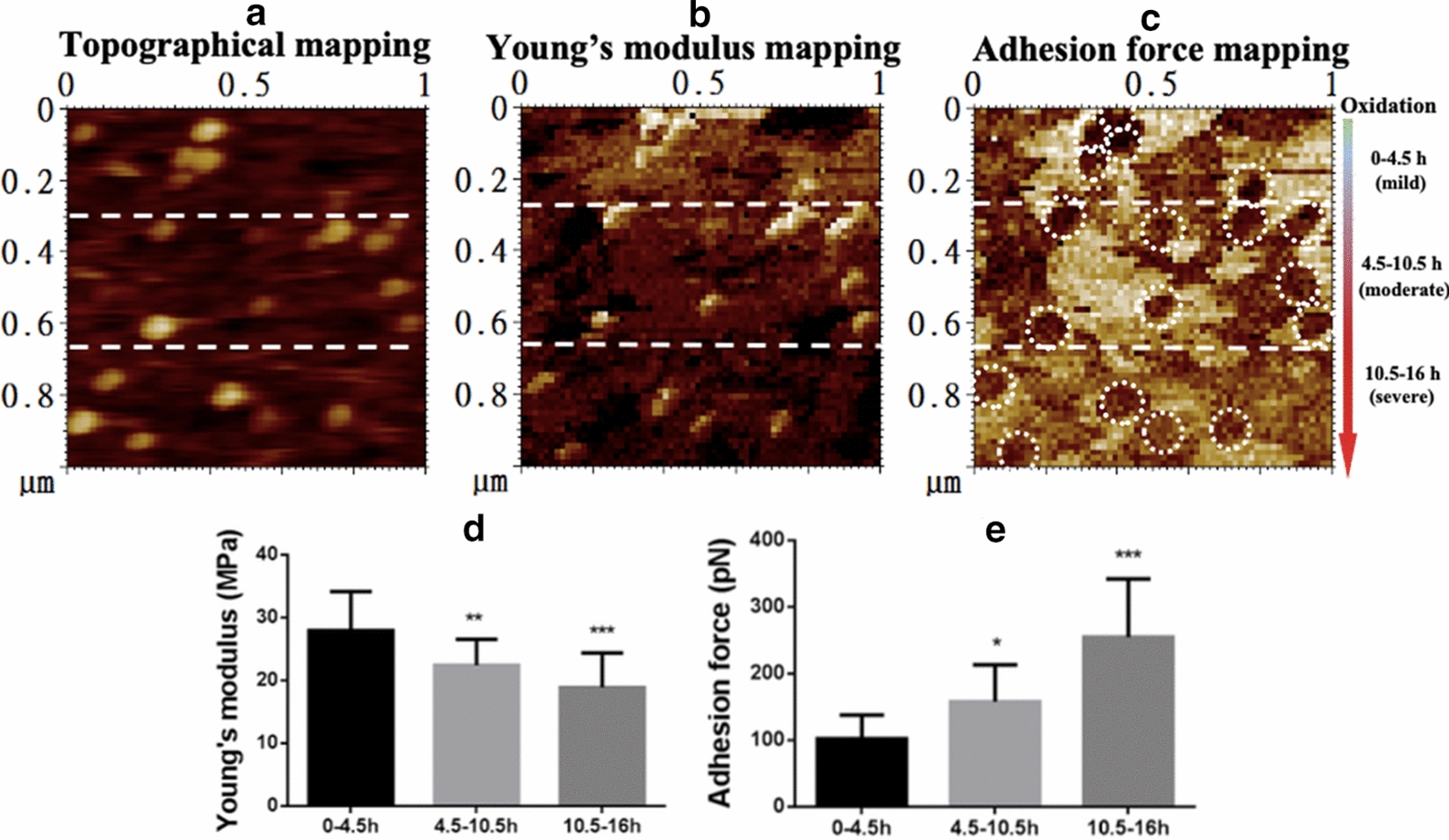
Dynamic observation of the effects of oxidation on the biomechanical properties of LDL particles. Native LDL particles pre-immobilized on functionalized mica were mixed with 5 µM copper sulphate on the imaging stage of atomic force microscope and imaged immediately. The LDL particles were oxidized during imaging which lasted for ~ 16 h. It means that the particles from top to bottom in the same image were oxidized to different extents (from mild to severe). For the convenience of quantification, the image was divided into three sections from top to bottom (as indicated by the dashed lines), representing the LDL particles oxidized for 0–4.5 h (no or mildly oxidized), 4.5–10.5 h (moderately oxidized), and 10.5–16 h (severely oxidized), respectively. **a** Topographical mapping of the LDL particles in a field of 1 µm × 1 µm. **b** Young’s modulus mapping of the same particles. **c** Adhesion force mapping of the same particles. The white dashed circles in the adhesion force images indicate individual particle-like patches corresponding to the oxLDL particles at the same locations in the topographical image. **d** Quantification of the average Young’s modulus of the particles in each section. **e** Quantification of the average adhesion force of the particles in each section

To further confirm the above result, a dynamic observation of LDL oxidation was performed. We let LDL oxidation and AFM imaging happen simultaneously. The whole process (both oxidation and imaging) lasted for approximately 16 h. Since the scan direction was from top to bottom during AFM imaging, the oxidation time became longer and longer (from 0 h up to 16 h) from top to bottom, in another word, the oxidation of LDL particles became severer from top to bottom on each image (Fig. 4a–c). From the Young’s modulus mapping (Fig. 4b), it could be clearly observed that the brightness of the particle-like patches from top to bottom changed from strong to week, implying the decrease in Young’s modulus of individual particles. For the convenience of quantification, the whole images were divided into three equal sections from top to bottom which represent different periods of oxidation time (i.e. 0–4.5 h, 4.5–10.5 h, and 10.5–16 h, respectively) or different oxidation degrees of oxLDL particles (i.e. mildly oxidized, moderately oxidized, and severely oxidized, respectively) (Fig. 4a–c). The Young’s modulus and adhesion force data were extracted from individual LDL/oxLDL particles in each section for statistical analysis. The data shows that longer oxidation time (or severer oxidation degree) caused lower average Young’s modulus (28.0 ± 6.2, 22.4 ± 4.1, and 18.9 ± 5.5 MPa for mildly, moderately, and severely oxidized LDL, respectively; Fig. 4d) and higher average adhesion force (102.1 ± 35.7, 157.9 ± 55.4, and 254.8 ± 87.3 pN for mildly, moderately, and severely oxidized LDL, respectively; Fig. 4e), confirming the abovementioned result.

Interestingly, we observed a phenomenon only in the dynamic experiments, that is, the surface of the substrate (functionalized mica) became softer (darker in color) from top to bottom (Fig. 4b). This phenomenon was not caused by artifact because it did not occur in the topographical image and the adhesion force image which was obtained simultaneously with the Young’s modulus image. A potential explanation is that the lipids released from LDL particles due to oxidation (in the dynamic experiments LDL oxidation and AFM imaging were performed simultaneously) adhered onto the substrate and made the surface/background softer.

## Discussion

### Technical improvements in comparison with previous AFM studies on LDL

The sizes of native LDL particles are heterogeneous, so are the AFM-measured sizes of LDL (please see a brief summary for the reported AFM-measured sizes of LDL and HDL in our previous study [5]). Using different batches of LDL as samples may get various AFM-measured sizes of LDL. Therefore, it may cause data deviation to compare the size of oxLDL derived from a batch of native LDL with the size of another batch of native LDL. In the current study, for better comparison, we utilized the same batch of native LDL as the source of oxidized LDL and as the control instead of using a different batch of native LDL as the control in each independent experiment.

Dynamic imaging/detection of the LDL oxidation process also is an advantage in our study. For example, LDL oxidation and AFM imaging were conducted simultaneously (e.g. Fig. 4) as a method for verifying the above quantitative result. It means that not only the same batch of native LDL but also the same sample was used (i.e. the same LDL particles were compared during oxidation) in each independent experiment which could improve the accuracy of results.

In previous studies on the biomechanical properties of LDL/oxLDL [5, 13, 14], a monolayer or even multilayer of LDL/oxLDL particles was detected by AFM. Another important improvement in the current study was that each of dispersedly distributed LDL/oxLDL particles in a topographical image could find a corresponding particle-like patch at the same location in a biomechanical image (Figs. 3 and 4). Therefore, we could precisely extract the data in the center of each particle-like patch in a biomechanical image for quantification and avoid the possibility of including the surroundings/background (e.g. the mica surfaces and nearby particles) into force extraction/calculation. This process also could promote the accuracy of the biomechanical data greatly.

### Effect of oxidation on the size of LDL particles

Previously, we have reported that oxLDL particles have a slightly smaller AFM-measured average diameter than native LDL particles (19.5 ± 4.6 nm and 19.1 ± 3.2 nm for native LDL and oxLDL, respectively, P > 0.05; both were commercially purchased) [11]. In the current study, the oxLDL was obtained in our lab by copper-induced oxidation of native LDL (Fig. 1). By comparing the LDL particles before and after oxidation, we confirmed the oxidation-induced decrease in size of LDL particles (Fig. 2). It is well understood that oxidation (particularly copper-induced oxidation) generally leads to loss of LDL lipid components (e.g. peroxides, aldehydes, lyso-PC, etc.) and even protein components (e.g. hydrolysis or fragmentation of apolipoprotein B-100). For example, Fig. 1b shows that oxidation induced a dramatic malondialdehyde (MDA) production from LDL particles in an oxidation time-dependent manner. The oxidation-induced partial component loss of native LDL particles might be responsible for the significant decrease in size of oxLDL particles.

### Effect of oxidation on the stiffness of LDL particles

Previously, only a few reports investigated the biomechanical properties of plasma lipoproteins or LDL particles by using AFM. In one of our previous studies [5], we detected that the average Young’s modulus of LDL particles is ~ 400 MPa (0.39 ± 0.15 GPa) which is much higher than the value (20.1 ± 1.8 MPa) measured in the current study. In the previous study, a monolayer of densely distributed LDL particles was utilized and individual LDL particles were surrounded/squished tightly by several LDL particles. It was highly possible that when an LDL particle was pressed down by an AFM probe the surrounding crowded LDL particles might exert an effect on the pressure resulting in the measurement of a relatively high Young’s modulus value.

Takeda et al. [13] reported that the elastic modulus (or Young’s modulus) of LDL particles ranges between 0.1 and 2 MPa with an average of ~ 1 MPa and that oxLDL particles have smaller elastic moduli [14]. Their average elastic moduli are more than 20-fold lower than the data in our current study (20.1 ± 1.8 MPa and 16.7 ± 2.6 MPa for LDL and oxLDL, respectively). Firstly, in their study, an indentation length of 20–30 nm from the LDL surface to the mica surface (i.e. the thickness of their LDL samples) was measured. In most previous AFM studies [5, 15–17], however, the average heights of individual LDL particles deposited on a substrate are generally less than 10 nm (e.g. 8.9 ± 1.9 nm at pH 7.4 in our current study). A thickness of 20–30 nm reflects that their samples probably were two or more layers of LDL particles but not a monolayer. Secondly, their elastic moduli were estimated from the region of the contact point to 6 nm of indentation, in another word, many values were excluded artificially. Finally, in all of the three previous studies including ours [5, 13, 14], an one-to-one relationship of individual particles between topographical mapping and force mapping could not be obtained. Therefore, we think that the Young’s modulus data in the current study is more accurate.

Our data (Figs. 3 and 4) show that oxidation induces the decrease in stiffness of LDL particles (i.e. oxidized LDL particles are softer than native LDL particles). At present, it is unclear what are the major contributors to the oxidation-induced decrease in stiffness of LDL. The average Young’s moduli of LDL/oxLDL particles in the current study (20.1 ± 1.8 MPa and 16.7 ± 2.6 MPa, respectively) are ~ 10-fold lower than those of some viral capsids with similar sizes to LDL, e.g. hepatitis B virus (an animal virus; 260–370 MPa) [18, 19] and cowpea chlorotic mottle virus (a plant virus; 140–280 MPa) [20, 21], at the same order of magnitude as the Young’s moduli (10–100 MPa) of various liposomes [22], slightly higher than that of bacteria (1–10 MPa) [23, 24], and much higher than that of eukaryotic cells (< 1 MPa and even down to 0.02 kPa) [25, 26]. It implies that LDL/oxLDL particles are softer than some viral capsids with similar sizes whereas have a stiffness similar to liposomes and are stiffer than prokaryotic (bacteria) or eukaryotic cells.

### Effect of oxidation on the stickiness of LDL particles

In our previous study [5], we also detected that the average adhesive force of native LDL particles in PBS (pH 7.4) is ~ 200 pN (0.19 ± 0.12 nN). In the current study, the average adhesive force of native LDL particles at pH 7.4 is 113 ± 25 pN which is a little lower than the previous result. In the previous study, the adhesive force data were randomly extracted from the whole monolayer of densely distributed LDL particles whereas in the present study the data were specifically extracted from the particle-like patches in the adhesion force images corresponding to the same locations of LDL/oxLDL particles in the topographical images. Therefore, we think that the current adhesive force value is more accurate. Moreover, interestingly, we also found that a 16-h oxidation could induce an approximately 2-fold increase (e.g. 1.7-fold from 113 ± 25 pN to 192 ± 50 pN in Fig. 3c or 2.5-fold from 102.1 ± 35.7 to 254.8 ± 87.3 pN in Fig. 4e) in average adhesive force of LDL particles. Although at present it is unclear why oxLDL particles have a higher adhesive force than LDL particles (the more negatively charged surface of oxLDL might be one of the contributors since it has been reported that surface charges of samples can influence the adhesive force detected by AFM [27, 28]), a relatively high adhesive force may be partially responsible for the physical adhesion of oxLDL particles onto extracellular matrix or cell surfaces, and therefore for their retention in atherosclerotic plaques.

### Implications from the effects of oxidation on the biophysical properties of LDL particles

In this study, we revealed that oxLDL particles are smaller, softer (lower in average Young’s modulus), and stickier (higher in average adhesive force) than native LDL particles. The smaller and softer properties can enable oxLDL particles easier than native LDL particles to pass through the tiny intercellular gaps of vascular endothelium and the meshes of the extracellular matrix resulting in an enhanced infiltration. On the other hand, the stickier property can enable oxLDL particles easier and tighter than native LDL particles to adhere on the endothelium and on the extracellular matrix, resulting in an enhanced adhesion/retention/accumulation of oxLDL than native LDL. The stickier property of oxLDL probably is able to partially explain why oxLDL is prone to self-aggregation. Therefore, the size and biomechanical properties of oxLDL particles are closely correlated with the initiation and progression of atherosclerosis.

Moreover, the cholesterol level/content of LDL (or LDL-C) has long been regarded as a major risk factor of atherosclerosis. The cholesterol level of non-HDL and the particle level/amount of native LDL (or its sub-fractions with different particle sizes) as risk factors of atherosclerosis have also attracted more and more attention. We hypothesize that the biomechanical properties (e.g. stiffness and/or stickiness) of LDL/oxLDL particles are also a potential risk factor of atherosclerosis. More in-depth studies will be needed for proving this hypothesis after the AFM-based biomechanical detection is widely applied in the future research.

## Conclusions

In this study, the effects of copper-induced oxidation on the size, stiffness, and stickiness of native LDL particles were investigated by AFM. To ensure the accuracy of results, the following manipulations were performed: (a) the same batch of native LDL was utilized both as the source of oxidized LDL and as the control for comparison in each independent experiment; (b) the LDL/oxLDL particles were immobilized on the substrate in a dispersedly distributed manner instead of a densely distributed manner (e.g. monolayer or multilayer); (c) the biomechanical data of each LDL/oxLDL particle was extracted from the center of the particle-like patch in the biomechanical image which corresponded to the same location of the LDL/oxLDL particle in the topographical image; (d) dynamic AFM evaluation of the LDL oxidation process (i.e. AFM imaging and LDL oxidation were carried out simultaneously) was utilized to validate the quantitative data. Our AFM data show that LDL oxidation could induce a significant decrease in size (average height and diameter) and stiffness (average Young’s modulus) but a significant increase in stickiness (average adhesion force). The smaller, softer, and stickier properties of oxLDL particles compared with native LDL particles partially explain the atherogenic role of oxLDL in the initiation and progression of atherosclerosis.

## Data Availability

All data generated or analyzed during this study are included in this article.
